# A modified scoring system to describe gross pathology in the rabbit model of tuberculosis

**DOI:** 10.1186/1471-2180-11-49

**Published:** 2011-03-04

**Authors:** Mandeep S Jassal, Gueno G Nedeltchev, Jonathan Osborne, William R Bishai

**Affiliations:** 1 Center for Tuberculosis Research, Johns Hopkins University School of Medicine, Baltimore, MD, 21231, USA; 2 Division of Pediatric Pulmonology, Johns Hopkins University School of Medicine, Baltimore, MD, 21287, USA

## Abstract

**Background:**

The rabbit model is an ideal means to study the pathogenesis of tuberculosis due to its semblance to the disease in humans. We have previously described the results using a bronchoscopic route of infection with live bacilli as a reliable means of generating lung cavities in sensitized rabbits. The role of sensitization in the development of disease outcomes has been well established in several animal models. We have described here the varying gross pathology that result from lack of sensitization with heat-killed *M. bovis *prior to high-dose bronchoscopic infection with live bacilli.

**Results:**

Rabbits lacking sensitization did not generate lung cavities, but instead formed solely a tuberculoid pneumonia that replaced the normal lung parenchyma in the area of infection. Extrapulmonary dissemination was seen in approximately equal frequency and distribution in both rabbit populations. Notable differences include the lack of intestinal lesions in non-sensitized rabbits likely due to the lack of ingestion of expectorated bacilli from cavitary lesions. The experiment also employed a modified scoring system developed initially in the primate model of tuberculosis to allow for the quantification of findings observed at necropsy.

**Conclusions:**

To date, no such scoring system has been employed in the rabbit model to describe gross pathology. The quantitative methodology would allow for rapid comparative analyses and standardization of thoracic and extrapulmonary pathology that could be evaluated for statistical significance. The aim is to use such a scoring system as the foundation for all future rabbit studies describing gross pathology at all stages in TB pathogenesis.

## Background

Tuberculosis (TB) remains a critical public health problem where 9.1 million incident cases were noted in 2006. Within this same time frame, greater than 1.5 million deaths had been attributed to TB [1]. Infection with *Mycobacterium tuberculosis *(*M. tb*.) most often occurs via the pulmonary route with varying intra- and extrapulmonary pathologies noted in humans [2,3]. Several animals have been studied to mirror TB disease pathology including mice, guinea pigs and rabbits [4,5]. Rabbits are particularly appealing given the similar immune response noted in this population of naturally-resistant animals [6,7].

We have developed a rabbit model that utilizes a bronchoscopic model of infection to reliably produce lung cavities. The model also demonstrated the unique extrapulmonary dissemination among animals infected with either *Mycobacterium bovis *(*M. bovis*) AF2122 or *M. bovis *Ravenel [8]. All rabbits that were infected were sensitized with heat-killed *M. bovis *to maximize the probability of cavity formation. The importance of sensitization in our experiment was based on classical studies by Wells and Lurie who demonstrated pulmonary cavities in rabbits pre-sensitized with heat-killed *M. bovis *and challenged with low-dose *M. bovis *[9]. Ratcliffe and Wells further expanded on the importance of sensitization when they noted cavity formation in rabbits that underwent low dose *M. bovis *infection and were subsequently infected with high-dose *M. bovis *[10]. Yamamura et al. had also elucidated the importance of sensitization when he published a series of studies that described the reliable production of lung cavities in 30-60 days. Only rabbits pre-sensitized at regular intervals with heat-killed *M. bovis *formed cavities when undergoing intrathoracic infection with live or heat-killed mycobacteria [11,12].

The present report explores the varying thoracic and extrapulmonary pathology secondary to sensitization using heat-killed *M. bovis *in the bronchoscopic model of infection. The primary aim was to determine if a modified scoring system, initially employed in the cynomolgus macaque model of tuberculosis, could be utilized to quantitatively depict and standardize the gross differences that exist on necropsy in two types of experimental rabbit populations [13]. Such a numerical means of description, which has never been performed in the rabbit model of tuberculosis, would allow for a rapid and reliable means of enhancing the description of TB disease pathogenesis. The quantitative intrapulmonary and extrapulmonary differences attributed to sensitization were validated against traditionally employed modalities of CFU counts and descriptive observations.

## Results

### Varying lung pathology based on sensitization status

Sensitized rabbits were injected at regular intervals using heat-killed *M. bovis *with all converting their tuberculin skin tests positive 25 days after the last sensitization injection (Table 1). Positive reactions were concluded if any measurable reaction was observed. Non-sensitized animals did not undergo skin testing prior to infection due to the lack of exposure to the sensitizing agent. Sensitized rabbits were observed for an average of 72 days (range = 50-98 days). The shortest time period of observation was in Rabbit Bo(S)4 and the longest elapsed time was in sensitized rabbit Bo(S)5. Non-sensitized rabbits were observed for an average of 55 days (range = 37-79).

**Table 1:** Bacillary infections and tuberculin skin test data in rabbit populations.

Sensitization Status	**Skin testing (mm**^**3**^)	Days of Infection Prior to Necropsy	Instilled Dose (CFU)
**Sensitized rabbits**			

AF1 (*M. bovis *AF2122)	1013 mm^3^	85	18,0000

AF2 (*M. bovis *AF2122)	748 mm^3^	90	18,0000

AF3 (*M. bovis *AF2122)	1507 mm^3^	50	18,0000

AF4 (*M. bovis *AF2122)	1761 mm^3^	58	18,0000

Bo(S)1 (*M. bovis *Ravenel)	1291 mm^3^	98	18,0000

Bo(S)2 (*M. bovis *Ravenel)	1482 mm^3^	57	18,0000

Bo(S)3 (*M. bovis *Ravenel)	1495 mm^3^	61	18,0000

Bo(S)4 (*M. bovis *Ravenel)	1245 mm^3^	64	18,0000

Bo(S)5 (*M. bovis *Ravenel)	1404 mm^3^	83	18,0000

**Non-sensitized rabbits**			

AF5 (*M. bovis *AF2122)	n/a	61	18,000

B1 (*M. bovis *Ravenel)	n/a	54	8000

B2 (*M. bovis *Ravenel)	n/a	55	8000

Bo1 (*M. bovis *Ravenel)	n/a	65	10000

Bo2 (*M. bovis *Ravenel)	n/a	63	10000

Bo3 (*M. bovis *Ravenel)	n/a	61	15000

Bo4 (*M. bovis *Ravenel)	n/a	62	10000

Two strains of *M. bovis *were utilized with similar pathologic endpoints observed in both non-sensitized and sensitized rabbits. Select sensitized rabbits were followed up to 100 days post-infection. Non-sensitized rabbits were observed up to 60 days after bronchoscopic infection. Intradermal skin testing was performed prior to infection on sensitized rabbits 25 days after the last sensitization injection to confirm successful acquisition of delayed-type hypersensitivity (DTH) immunity. Non-sensitized animals did not undergo skin testing prior to infection due to the lack of exposure to the sensitizing agent. Varying dosages and duration of infection were seen in the sensitized and non-sensitized rabbits based on initial experimental objectives prior to the application of this retrospective study.

**Figure 1:**
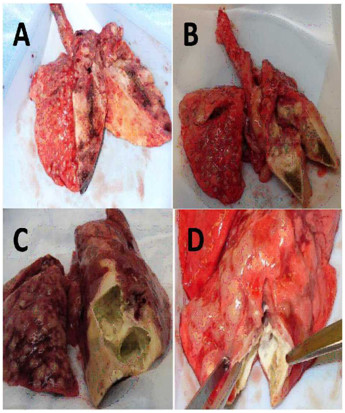
**Gross pathology of select lung specimens on necropsy**. Panel A & B represent non-sensitized rabbits B1 and AF5, respectively. Neither display a discernable cavitary lesion but complete effacement of the right lower lung parenchyma by a tuberculoid pneumonia is present. Both had numerous bilateral granulomas of the visceral surface of the lung. Panel C & D include sensitized rabbits Bo(S)1 and AF1, respectively. Both rabbits display cavitary formation in the site of bronchoscopic infection of the right lower lobe. Similar gross pathology exists in the contralateral lungs in sensitized and non-sensitized rabbits.

All sensitized *M. bovis *AF2122 and Ravenel infected rabbits yielded cavity formation at the site of bronchoscopic infection (Figure 1). The sole exception was Rabbit AF4 which formed multiple coalescing granulomas at the infection site. Cavity walls possessed various amounts of necrosis and fibrosis. Non-sensitized animals did not develop any lung cavities despite over 50 days of observation. The right lower lobe contained caseous material in all non-sensitized rabbits but no signs of liquefaction were observed.

A tuberculoid pneumonia characterized by complete destruction of the lung parenchyma by the infectious process was isolated to the right lower and middle lung lobes in both rabbit populations (Figure 1). The right ipsilateral lung developed multiple granulomas distributed throughout the visceral surface. The contralateral lung also yielded similar formations of numerous granulomas on its surface regardless of sensitization status. Multiple granulomas, of various sizes, were appreciated on all lung lobe segments in both populations of rabbits. A larger proportion (> 10 granulomas) were more frequently noted on the ipsilateral surface. Dissection into the lung parenchymal structure in the right upper and contralateral lungs yielded no pneumonic process.

**Figure 2:**
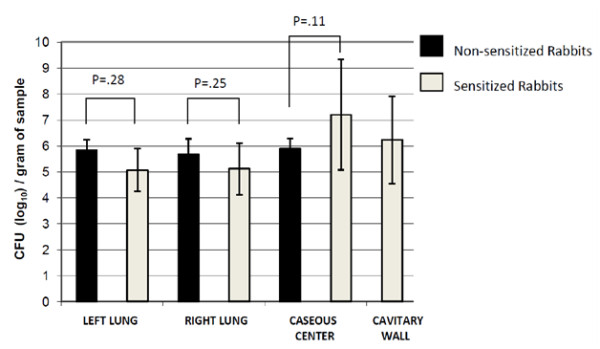
**Mean pulmonary CFU counts in sensitized and non-sensitized rabbits**. At necropsy, multiple samples of the right lung, left lung, caseous center and cavitary wall were obtained. The log CFU count/gram of tissue was determined after homogenization and plating dilutions. Non-sensitized rabbits had greater CFUs in the lung parenchyma bilaterally. Non-cavitary caseous centers in non-sensitized rabbits had fewer CFUs compared to sensitized animals. Cavitary lesions were uniquely observed in sensitized rabbits. P values, for which none achieved significance, are based on average CFU counts of sensitized versus non-sensitized rabbits at each comparable intrathoracic site. Error bars represent standard error of the mean.

Mean pulmonary CFU counts reveal the largest bacterial load in the caseous lesions found at the site of bronchoscopic infection (Figure 2). Sensitized rabbits had greater than 1.5 log bacterial load in the caseous contents compared to non-sensitized animals. Cavitary wall CFUs were apparent in only sensitized rabbits and yielded one log fewer bacilli as compared to liquefied cavitary caseous contents. Ipsilateral and contralateral lung CFUs were higher by approximately one-half log in non-sensitized rabbits. Varying lung granuloma sizes and numbers among both sensitized and non-sensitized rabbits did not appear to correlate with greater bacillary load. Only the presence of cavitary lesions were indicative of a greater number of bacilli.

### Relative uniformity of extrapulmonary dissemination in *M. bovis *infected rabbits

**Figure 3:**
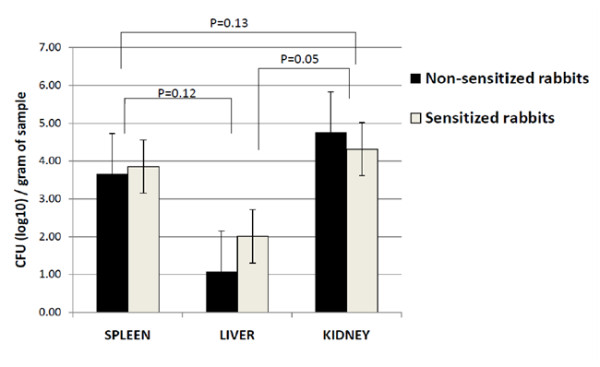
**Mean extrapulmonary CFU counts in sensitized and non-sensitized rabbits**. At necropsy, samples of the spleen, kidney and liver were obtained. The log CFU count/gram of tissue was determined after homogenization and plating dilutions. Sensitized rabbits had greater CFUs in the spleen and liver. Non-sensitized rabbits had approximately half log more CFUs as compared to their sensitized counterparts. P values are based on average CFU counts among both rabbit populations at each extrapulmonary site and compared to other selected areas. Error bars represent standard error of the mean.

As noted in previous published work by Nedeltchev et al, *M. bovis *uniquely disseminates to extrapulmonary locations as compared to *M. tb *[8]. All rabbits in this study also displayed extrapulmonary dissemination with detectable CFUs most prominently noted in the spleen, liver and kidney. No cavitary formation was appreciated in any extrathoracic organ. Gross pathology revealed granulomas on each kidney of both sensitized and non-sensitized rabbits. Corresponding with the greater observed kidney pathology were more detectable CFUs (Figure 3). The kidneys of non-sensitized rabbits had approximately 0.3 log more CFUs. Splenic lesions were noted in three sensitized rabbits (AF1, AF4, Bo(S)3) and one non-sensitized rabbits (Bo1). The mean spleen CFUs were slightly higher in rabbits undergoing sensitization. Fewer splenic CFUs were noted, though not significant (p > 0.1), when compared to kidney CFUs in non-sensitized rabbits. This observation is contrary to the findings in our previously published work [8]. Spleen counts were noted in prior studies to have the highest amount of detectable extrapulmonary bacillary load due likely to its role as a key reticuloendothelial organ. Differences between observed CFUs and gross pathology were noted in the liver where detectable CFUs could be found in both rabbit populations but tuberculomas were not observed at necropsy. The liver had the lowest CFU counts among all observed organs and tissues. No involvement of the cecum was noted in non-sensitized rabbits which would correspond with the lack of cavitary formation. Granulomas of the appendix were noted in all sensitized rabbits with the exceptions of AF1 and AF2.

### Correlation of grossly observed outcomes with numeric scoring system

A numerical scoring system was initiated to provide a consistent means to evaluate gross pathology (Additional File 1). The scoring system was based on the methodology utilized by Lin et al. for the cynomolgus macaque model [13]. Based on detailed photographs obtained at necropsy, rabbits were assigned a quantitative measure of their disease pathology. The maximum score assigned was 50. The organs or tissues chosen were determined from previous studies that utilized descriptions of each respective site as a means of characterizing disease outcomes [8]. Lesions from each lobe were enumerated based on the number of granulomas or extent of tuberculous pneumonia. The right lower lobe was of particular focus with the description of a cavitary process at the site of infection being assigned the greatest numeric score (total = 10). A lung cavity was given the highest score based on its primary significance on the ultimate mortality and morbidity of the animal. Previous work by Nedeltchev et al. had shown that the bronchoscopic route of infection was ideally utilized for generating the maximum amount of intra and extrapulmonary pathology due to its ability to consistently reproduce lung cavities [8]. Pleural lesions were characterized by either the absence or presence of adhesions or parietal granulomas which are often observed in the context of a bronchopleural fistula. Extrapulmonary dissemination was quantified by the presence and number of granulomas in the liver, spleen, appendix and kidneys. The sole lymph node sites evaluated included mediastinal and thymic tissues. The mediastinal and thymic tissues were classified together due to the difficulty of individually separating these closely located anatomic sites.

**Figure 4:**
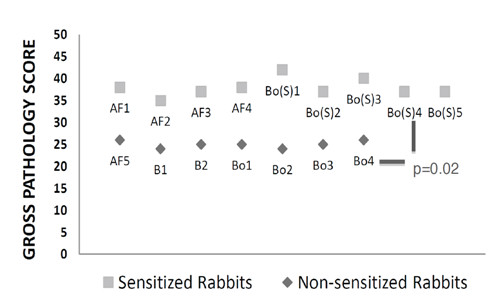
**Gross pathology scoring system in sensitized and non-sensitized rabbits**. Additional File 1 constitutes the details of the scoring system employed. All evaluable rabbits were analyzed with a maximum possible score of 50. Sensitized rabbits had significantly greater scores compared to non-sensitized animals.

The intrapulmonary spectrum of disease was greater in sensitized rabbits which uniquely developed lung cavities (Figure 4). All sensitized rabbits had greater total scores invariably due to the assigned numerical importance of these lesions. Rabbits Bo(S)1 and Bo(S)3 had the highest total scores in sensitized rabbits due to the observed extrapulmonary granulomas in the spleen and appendix. The enumeration of extrapulmonary pathology was approximately equivalent in both species. Discrepancies between observed CFUs and gross pathology were notable in the liver where detectable CFUs could be found in both rabbit populations but tuberculomas could not discerned at necropsy. Statistical significance was achieved (p = .02) when comparing the mean gross pathology scores among the two rabbit populations. The observed necropsy findings and CFU counts appear to correlate with the employed numeric scoring system.

## Discussion

The primary purpose of this paper was to explore the validity of a modified scoring system, which was initially developed for the cynomolgus macaque model of tuberculosis, to be employed in disease outcomes in sensitized and non-sensitized rabbits. The scoring system correlated well with the observed differences noted in our two experimental population of animals. Sensitized rabbits uniquely generated lung cavity formation when challenged with live *M. bovis *bronchoscopic infection. Non-sensitized rabbits consistently generated significant bilateral granulomas with a focal tuberculoid pneumonia in the right lower lung area of infection. Multiple granulomas, of varying sizes, were appreciated in all lung lobes with the greatest frequency appreciated in the ipsilateral site of infection. Diffuse extrapulmonary dissemination was seen in all rabbits with minimal intrasubject variability noted.

The importance of sensitization in the development of cavitary lesions was best elucidated by the work of Yamamura et al [11,12]. Sensitization was undertaken using heat-killed *M. bovis *suspended in Freund's adjuvant, paraffin oil and anhydrous lanolin. Rabbits were injected subcutaneously 4 to 5 times with heat-killed *M. bovis *at intervals of 5 to 7 days. After one month from the first sensitization, rabbits were infected with a live *M. bovis *via intrathoracic injection. With this methodology, lung cavities developed between 30-60 days post-infection with reproducibility. Pulmonary cavities were also produced post-sensitization when either whole heat-killed bacilli, paraffin-oil extracts of heat-killed bacilli or mycobacterial proteolipid components were utilized [11,14]. The researchers also demonstrated that desensitization to mycobacterial lipoprotein could inhibit the lung cavity formation [15].

The significant clinical outcomes noted with sensitization is intriguing given the numerous instances in which sensitization may occur in the human setting. Humans may be sensitized by being exposed either repeatedly to *M. tb*. in their environment or immunization with the Bacille Calmette-Guérin (BCG) vaccine [16,17]. The instances in which resulting cavitary formation occurs is critical since this is the key means of disease transmission [18]. This paradigm may also hold true for nontuberculous mycobacteria which has been attributed to increasing cases of human disease [19].

However, the need for sensitization in developing lung cavities is not absolute given the work by Converse and Dannenberg who had developed an aerosol model that reliably produced cavities in non-sensitized rabbits. Moderately low doses of *M. bovis *(10^2^-10^3 ^CFUs) yielded lung cavities in 9 of 12 rabbits. Higher doses *M. bovis *infections (10^3^-10^4 ^CFUs) generated cavitary lesions in all 6 animals studied after 5 weeks of observation [20].

Lung cavities seen in this study in sensitized M. bovis-infected rabbits showed central caseation and liquefaction that were also noted in our prior work [8]. All rabbits showed a fragile, non-homogenous caseum. Cavity walls had a variable amount of necrosis and fibrosis. CFU counts had expectedly shown the largest number of bacilli in the cavitary center and wall with greater than 6 log of bacilli yielded at each site. Our previous work had also noted that sensitized rabbits had generated diffuse intrapulmonary dissemination with multiple bilateral granulomas. Non-sensitized rabbits had produced similar pathology with diffuse granulomas appreciated in all but the focal area of bronchoscopic infection. In the right lower lung lobe, all non-sensitized rabbits had their parenchymal architecture replaced by a tuberculoid pneumonia. In select non-sensitized rabbits, the right lower region showed a caseating lesion that did not undergo liquefaction. These caseous areas yielded greater CFUs than any other evaluable anatomical site. The increased amount of bacilli in these central areas are to be expected given the host's limited immune response characterized by reduced macrophage function and entry. Human pulmonary cavities can generate approximately 10^7^-10^9 ^bacilli in their liquefied caseum [21,22].

Our previous work also described a key difference between *M. bovis *and *M. tb*. bronchoscopic infection where extrapulmonary dissemination was noted more prominently in *M. bovis *infected rabbits [8]. However, classical studies that utilized an intravenous route of infection displayed extrapulmonary dissemination in both non-sensitized *M. tb*. and *M. bovis *[23]. Both species showed spread to the spleen, liver and kidneys. But only rabbits infected with M. bovis-infections showed continued disease pathogenesis that could not be controlled by the rabbit's innate immune system. The current experiment described extrapulmonary dissemination in both non-sensitized and sensitized rabbits. The kidneys displayed the greatest amount of CFUs with both animal populations having approximately one log greater bacilli compared to the spleen. Splenic CFUs were notably fewer (p > 0.1) which is contrary to our expectations given the spleen's role as a key reticuloendothelial organ [8]. We suspect that the difference in CFUs may have been due to the selected regions used for plating culturable splenic and kidney CFUs. If full tissues specimens had been utilized, then the results may have been comparable in both studies. Non-sensitized rabbits in general had also fewer cecal lesions that were likely attributable to the absence of pulmonary cavity formation. Rabbits with cavitary lesions were noted to have approximately 1 log greater CFU in sampled gastrointestinal sites. Expectorated bacilli from lung cavities are suspected to be swallowed by the rabbits to yield intestinal lesions [20]. However, the possibility of intestinal lesions being derived from extrapulmonary dissemination via the bloodstream or contiguous spread via the retroperitoneal/gastrointestinal space must also be considered.

This experiment further validates the bronchoscopic infection methodology and expands our understanding of TB pathogenesis in the rabbit model. The classically utilized descriptions of disease outcomes including gross pathology and microbiology appear to correlate with our adapted scoring system. This quantitative approach has allowed us a statistical means by which to classify disease outcomes. Differences in total gross pathology scores had been noted on necropsy in this study between sensitized and non-sensitized rabbits. The significant findings were largely attributable to the unique formation of cavitary lesions on gross pathology which is supported by subsequent enumeration of CFUs. With the sole exception of notable CFUs in the liver where no tuberculomas were seen on necropsy, the observed gross pathology score on necropsy was a reliable means by which to base disease outcomes.

Our scoring system is modified from an earlier one published by Lin et al. for the cynomolgus macaque model of TB. Our numerical system allows for a greater score to cavitary disease (a key endpoint in our bronchoscopic approach) and eliminates select pathology that is not of immediate relevance to the rabbit model. Clinical based outcomes, specifically signs of respiratory distress, were not added to our system but appeared to be a reliable tool of disease progression. However, temperature and weight changes obtained on a biweekly basis did not appear to differ significantly between our two populations of rabbits. Our employed methodology would ideally be used with CFU data with the benefit of providing rapid quantitative results at necropsy. Immediate analyses of the disease process could enhance the evaluation of vaccines or drug studies.

Limitations in the work include the use of the gross scoring system undertaken in a retrospective manner. The scoring system was adopted after necropsy had been undertaken. We had utilized a retrospective design by analyzing multiple angle photos and detailed notes to determine pathology scores. Future prospective usage of the scoring system may include variables not utilized in our study but originally included in the Lin et al. model. These include lung granuloma sizes, additional lymph nodes sites and non-abdominal extrapulmonary organs. A second limitation is the lack of immunologic and molecular based assays as an alternative means to validate our scoring system. Sharpe et al. also has noted in the rhesus macaque model of TB that MR imaging is an accurate and simple means to standardize disease outcomes [24]. Future experiments may be able to incorporate imaging as another quantitative approach to enhance our methodology. A final limitation is the varying time of observation from infection to necropsy and differing dosage of infection in non-sensitized versus sensitized rabbits. This disparity in time was largely due to the different objectives for the original experiments that the rabbits had been studied. The earlier period of necropsy due to respiratory distress in non-sensitized rabbits may not have been due to simply progressive gross pathology but a product of greater sedation and frequent endotracheal intubation required for experimentation. Future characterization of disease pathology may differ in non-sensitized rabbits if observed for longer time intervals with less frequent airway manipulations. Longer durations of infection may increase bacterial loads and alter the gross pathology which underlies our scoring system. Standardization of the dosage of infection may also allow for a more accurate interpretation of the differences in pathology between the two populations of rabbits. Moreover, upcoming experiments could use different sensitizing agents to determine if qualitative and quantitative differences could be observed on necropsy. The use of various sensitization compounds could be insightful into the role of the host's immune response to disease outcomes.

## Conclusions

The quantitative scoring system adapted for the rabbit model of tuberculosis may be a valuable tool for future animal studies to standardize observable outcomes of disease. The numeric-based methodology may allow for a reliable and rapid means of detecting the varying pathology seen in our animal experiments. Sensitization using heat-killed *M. bovis *uniformly promotes the development of cavitary formation when rabbits are exposed to high dose infection using live M. bovis. Lung pathology in non-sensitized rabbits consistently yielded a tuberculoid pneumonia at the site of bronchoscopic infection. The contralateral lung formed multiple granulomas and showed a similar pathology in both animal populations. Both sensitized and non-sensitized rabbits displayed extrapulmonary dissemination with the most notable difference being the lack of intestinal lesions in non-sensitized rabbits. The scoring system correlated well with the described findings at necropsy and may be used in a modified form in the future to enhance our studies in the rabbit model of tuberculosis.

## Methods

### Microrganisms

Cultures of *M. bovis *Ravenel and *M. bovis *AF2122 were prepared by thawing frozen stock aliquots for bronchoscopic infection. Mycobacteria were grown in 7H9 Middlebrook liquid media supplemented with oleic acid albumin, dextrose and catalase (OADC, Becton Dickenson, Inc., Sparks, MD), 0.5% glycerol and 0.05% Tween 80 and cyclohexamide (100 μg/mL). The glycerol-containing medium, as opposed to a pyruvate carbon source, was not found to limit the growth of *M. bovis *strains.

### Animals and infection

Sixteen pathogen-free outbred New Zealand White (2.5 to 3.5 kg) rabbits were obtained from Covance Research Products, Inc (Denver, PA). Animals were maintained in standard cages under biosafety-level 3 conditions. All animals were maintained in accordance with protocols approved by the Institutional Animal Care and Use Committees of Johns Hopkins University. One *M. bovis *AF2122 and six *M. bovis *Ravenel rabbits did not undergo sensitization. Sensitization had been undertaken in four *M. bovis *AF21122 and five *M. bovis *Ravenel rabbits. Rabbits that underwent sensitization received 5 subcutaneous injections with 10^7 ^heat-killed *M. bovis *in incomplete

Freund adjuvant (IFA) performed 3-4 days apart. An intradermal skin test with 0.1 cc of Old Tuberculin (Synbiotics Corp, Kansas City, MO) was given 25 days after the last sensitization injection in all sensitized animals. Skin testing was performed in the midsection of the flank region. The tuberculin reaction was read 48-72 hours later to confirm successful acquisition of delayed-type hypersensitivity (DTH) immunity with measurements being taken in two dimensions with a skin fold thickness and the results calculated using the formula for the volume of an oval spheroid. A successful reaction was concluded if any measurable reaction was observed. Non-sensitized rabbits did not undergo skin testing prior to infection given the assumption that intradermal skin testing should be non-reactive in this pathogen-free population. Rabbits were bronchoscopically infected with either *M. bovis *subspecies and tuberculin reaction was measured in sensitized animals after 40 days post-infection. Anesthesia induced by Xylazine (5-10 mg/kg) and Ketamine (15-25 mg/kg). Yohimbine (0.1-0.2 mg/kg) was utilized for reversing excessive sedation. A 3.0 flexible Pentax FB-8V pediatric bronchoscope (Pentax Medical Company, Montvale, NJ) was wedged into the right basal lobe of the lung. A total of 0.3 mL of bacilli suspension containing from 8000-18000 CFU was delivered via the bronchoscope insertion port.

### Clinical assessment

After infection, the rabbits were monitored twice weekly for clinical appearance, weight and rectal temperature.

### Necropsy

Rabbits were observed for a minimum of 50 days after infection in both non-sensitized and sensitized animals. Sensitized rabbits were in general observed for longer time periods up to a maximum of 105 days post-infection. Criteria to be euthanatized included signs of respiratory distress (dyspnea) and/or significant loss of weight (150-200 g). Rabbits were euthanized with intravenous Euthasol (Virbac Corporation, Fort Worth, TX). At necropsy, samples from the lungs and extrapulmonary sites were obtained. Cavity specimens that represented the primary lesion included (a) lumen contents, (b) wall and (c) surrounding inflammatory tissue. Grossly visible secondary lesions were noted of the ipsilateral lung, contralateral lung and extrapulmonary sites. Extrapulmonary locations included (a) lymph nodes (mediastinal), (b) spleen, (c) liver, (d) kidney (bilateral), (e) appendix.

### Determination of bacterial counts

Colony-forming unit (CFU) counts were measured from all pre-determined pulmonary and extrapulmonary sites. Tissue samples were selected based on areas which showed significant gross pathology (i.e. granulomas, cavitary regions, etc.). These sites were homogenized and aliquots were plated on BD (Becton Dickenson, Inc., Sparks, MD) Middlebrook 7H11 selective agar supplemented with Polymyxin B (200,000 units), Carbenicillin (50.0 mg), Amphotericin B (10.0 mg), Trimethoprim Lactate (20.0 mg) to hinder bacterial and fungal overgrowth. CFU counts were enumerated on day 14, 21 and day 28.

### Scoring of gross pathology

A gross pathology scoring sheet was developed to enumerate the gross pathology seen at necropsy (Additional File 1). The sheet was based upon an earlier published scoring sheet in the cynomolgus maqaque model by Lin and colleagues [13]. Grossly visible lesions from all lung lobes and extrapulmonary sites were described and enumerated. The total score was determined by adding all subtotal numbers assigned to each evaluable anatomic site. Standard descriptive strategies were also employed to document disease burden at necropsy and compared to the developed scoring system.

### Statistical analysis

Data are reported as mean values unless otherwise stated. Mean quantitative scores based on gross pathology were compared via non-parametric analyses by Mann-Whitney U test. Mean paired values of thoracic/extrapulmonary CFUs were summed and compared by paired t-test analyses. Tissue CFUs in each rabbit population were paired, regardless of sensitization status, during comparative tissue analyses. The level of significance was set at P < 0.10.

## Authors' contributions

MJ, GN and WB conceived and designed the experiments. MJ, GN and JO performed the experiments. MJ, GN and WB analyzed the data. MJ, GN and WB wrote the manuscript. All authors read and approved the final manuscript.

## Supplementary Material

Additional file 1 **Gross Scoring System Employed for the Rabbit of Tuberculosis**. A scoring sheet was developed to enumerate the gross pathology seen at necropsy. Visible lesions from all lung lobes and extrapulmonary sites were described and enumerated (maximum possible score of 50). The total score was determined by adding all subtotal numbers assigned to each evaluable anatomic site. Click here for file
