# Supplementary figures and images for: A modified scoring system to describe gross pathology in the rabbit model of tuberculosis
Source: BMC Microbiol. 2011 Mar 4;11:49. doi: 10.1186/1471-2180-11-49 (PMC3058006; doi:10.1186/1471-2180-11-49)

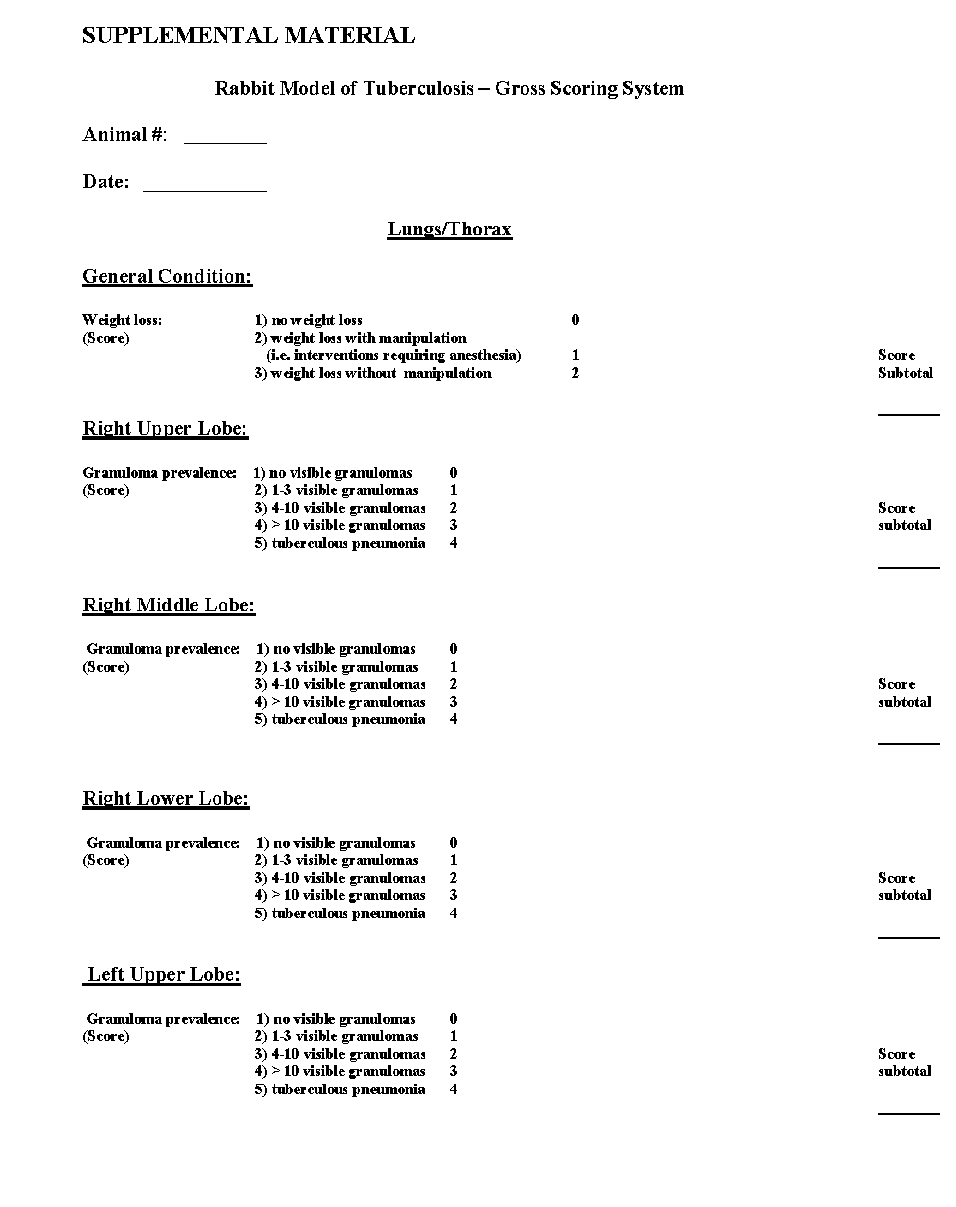


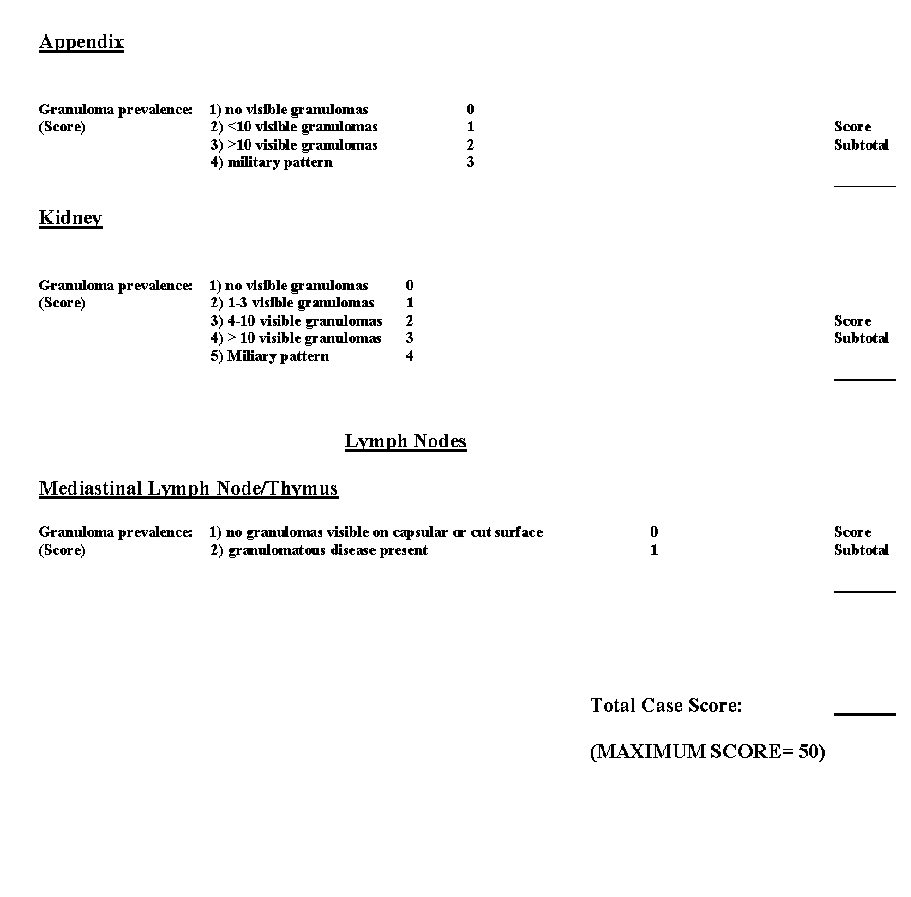

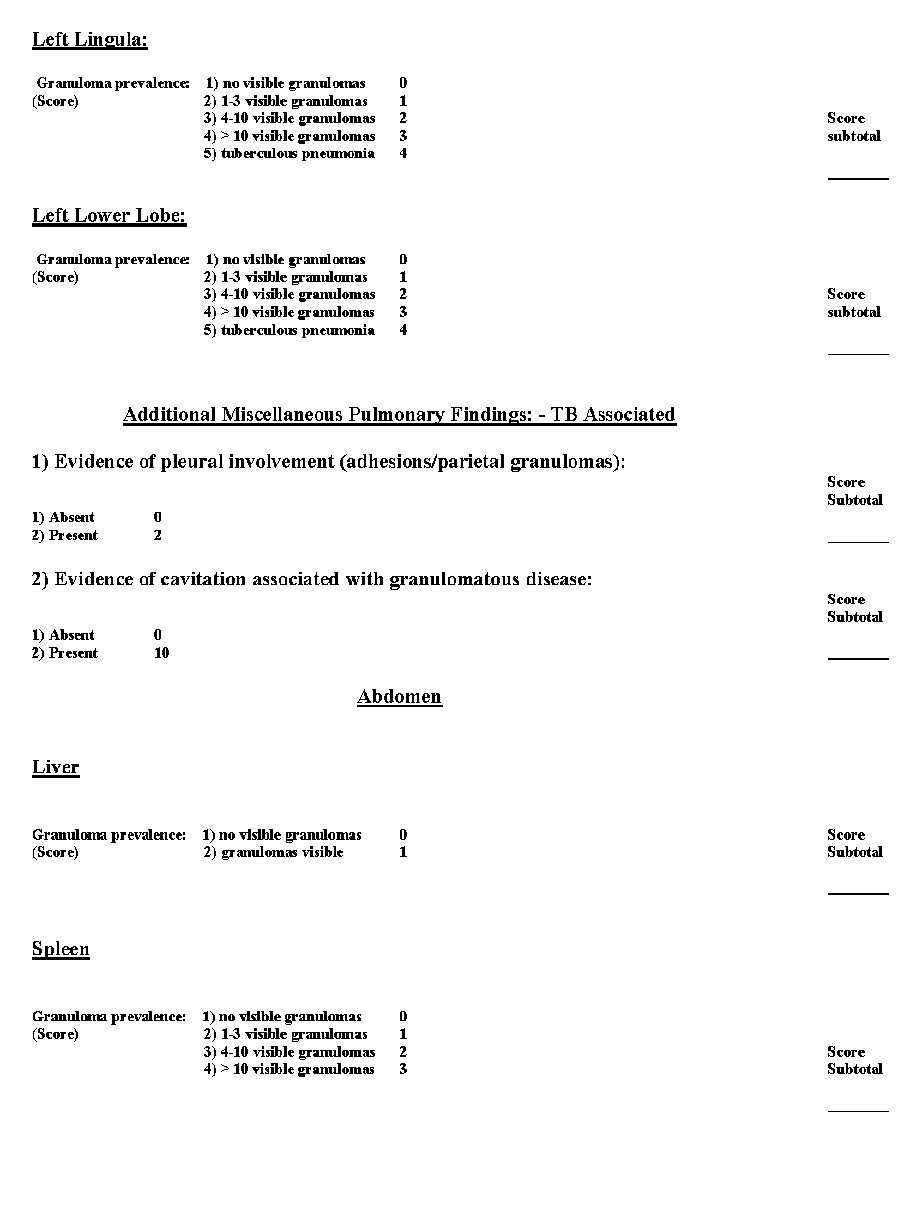

Supplement: Additional file 1 — Gross Scoring System Employed for the Rabbit of Tuberculosis. A scoring sheet was developed to enumerate the gross pathology seen at necropsy. Visible lesions from all lung lobes and extrapulmonary sites were described and enumerated (maximum possible score of 50). The total score was determined by adding all subtotal numbers assigned to each evaluable anatomic site. [file 1471-2180-11-49-S1.DOCX]
